# The Outcomes of Thoracic Endovascular Aortic Repair in Japan in 2017: A Report from the Japanese Committee for Stentgraft Management

**DOI:** 10.3400/avd.ar.20-00160

**Published:** 2021-09-25

**Authors:** Katsuyuki Hoshina, Kimihiro Komori, Hiraku Kumamaru, Hideyuki Shimizu

**Affiliations:** 1 Department of Vascular Surgery, Graduate School of Medicine, The University of Tokyo, Tokyo, Japan; 2 Division of Vascular Surgery, Department of Surgery, Nagoya University Graduate School of Medicine, Nagoya, Aichi, Japan; 3 Department of Healthcare Quality Assessment, School of Public Health, Graduate School of Medicine, The University of Tokyo, Tokyo, Japan; 4 Department of Cardiovascular Surgery, Keio University, Tokyo, Japan

**Keywords:** thoracic endovascular repair, thoracic aortic aneurysm, aortic dissection, annual report, the Japanese Committee for Stentgraft Management (JACSM)

## Introduction

Thoracic endovascular aortic repair (TEVAR) was initially used in treating descending thoracic aortic aneurysms back in 1992.^1)^ It was then approved by the United States Food and Drug Administration in 2005. Since then, TEVAR has been adopted worldwide and has been improved dramatically, both procedurally and in terms of available materials. In Japan, TEVAR is identified as the standard treatment for descending thoracic aneurysms.^2)^ The emergence of fenestrated stent grafts to better fit the angulated morphology of the thoracic arch and of the lower profile and the 2015 approval of TEVAR for aortic dissection have led to an increase in the annual number of TEVAR procedures performed in Japan. However, the outcomes of TEVAR are yet to be evaluated in a large cohort.

Thus, in this study, we aim to report the number of TEVAR procedures conducted in Japan in 2017, including its mortality and complication rates, by using the data from the Japanese Committee for Stentgraft Management (JACSM) nationwide registry; this data covers the outcomes of nearly all of the stent grafts shipped to Japan.^3,4)^

### JACSM and database

The JACSM nationwide registry, including its foundations, structure, and quality control, has already been previously described in detail.^3,4)^ The JACSM, established in December 2006, is composed of 10 societies related to endovascular treatment and determined the practical standards for institutions and for practicing and supervising surgeons. Participating institutions were obligated to report their data on endovascular aneurysm repair (EVAR) and TEVAR, using a web-based case-registry form (http://www.stentgraft.jp/).

The information collected as regards TEVAR included preoperative information, anatomical factors evaluated during operative planning, and data obtained in the immediate postoperative period, at discharge, at 6 months postoperative, and at each year after that. As of 2015, 366 institutes in Japan had been certified for TEVAR by the JACSM.

This registry was conducted in accordance to the principles of the Declaration of Helsinki, the International Conference on Harmonization, and Good Clinical Practice guidelines. The use of registry data was approved by the Institutional Review Board of the University of Tokyo Hospital (approval number: 2019306NI).

## Materials and Methods

### Groups and categories

**Figure 1:**
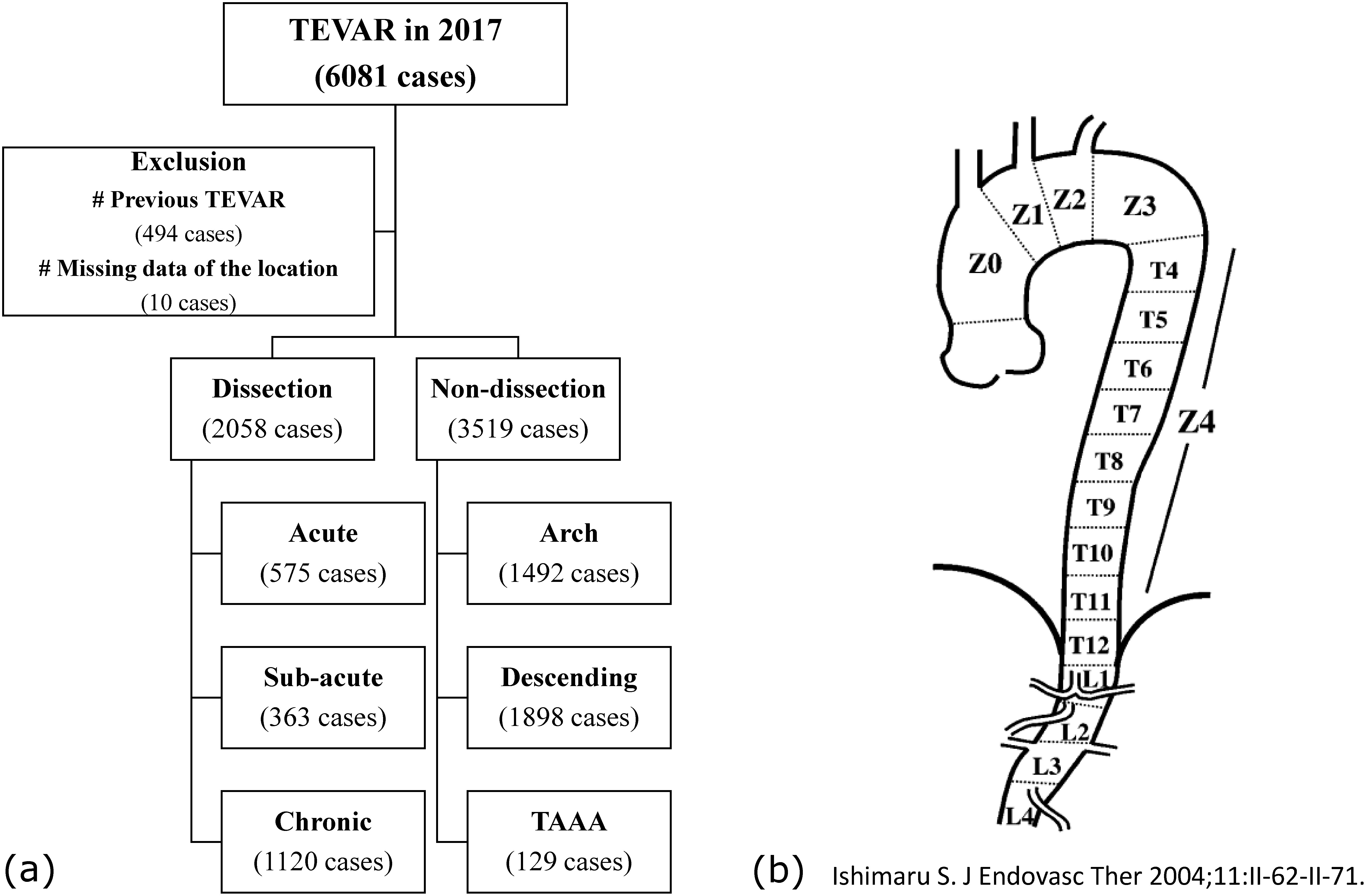
Fig. 1 Patients included in this study and the definition of the landing zone. (**a**) Flow chart of patient selection. (**b**) Definition of the landing zone (Ishimaru criteria).

Between January 2017 and December 2017, around 6,081 patients who underwent TEVAR were registered. The patients were excluded from this report if they had a history of TEVAR (n=494) or missing data regarding stent graft location (n=10). The final analysis included 5,577 patients (**Fig. 1a**). The patients were then divided into two groups: dissection (n=2,058) and non-dissection (n=3,519). Patients in the dissection group were further categorized as acute (TEVAR within 2 weeks of onset; n=575), sub-acute (TEVAR between 2 weeks and 2 months of onset; n=363), and chronic (TEVAR more than 2 months after onset; n=1,120). Meanwhile, patients in the non-dissection group were categorized according to the stent graft placement (Ishimaru criteria: **Fig. 1b**): arch (zone 0 to 2; n=1,492), descending (zone 3 to Th 12; n=1,898), and thoracoabdominal (TAA A) (L1 and distal; n=129) (**Fig. 1a**).

### Type of data collected

Data regarding patient age, sex, rupture, pathogenesis, comorbidities, renal function, condition of the proximal fixation, and preoperative aneurysm diameter were collected from the database for this report. Patient comorbidities included chronic obstructive pulmonary disease, hypertension (with medication), cerebrovascular infarction or hemorrhage, liver dysfunction, hemodialysis, carotid artery disease (≥75% stenosis), coronary heart disease with a history of intervention, history of thoracic surgery, history of abdominal surgery, history of thoracotomy, and Marfan syndrome. Renal function was then determined using serum creatinine level and estimated glomerular filtration rate. The diameter and landing length of the proximal fixation were then categorized in 5-mm increments. The diameters of aortic aneurysms categorized as “saccular” were specifically demonstrated. For the dissection group, data regarding the Stanford classification, complications related to aortic dissection (aortic dilatation, impending rupture, rupture, and malperfusion), and conditions of the dissected lumen (double barrel, thrombosed, or ulcer-like projection) were collected.

### Operative procedures

The type of stent grafts and anesthesia, proximal landing condition, occluded aortic branches, bypasses, and additional or emergent procedures (within 24 h after admission) can vary, depending on the discretion of the treating physician.

### Outcomes

The intraoperative rates of mortality, vascular injury, rupture, or endoleak and the intraoperative radiation dose were reported. Postoperative mortality or adverse events (e.g., migration, endoleak, thromboembolism, renal insufficiency, hemodialysis, cerebrovascular damage, paraplegia, multiple organ failure, and aneurysm rupture) before discharge from the hospital were also noted.

Categorical variables were presented as numbers and percentages, whereas continuous variables were presented as means±standard deviations. The duration of hospitalization after the operation is presented as median with interquartile range.

## Results

### Patient demographics

The mean patient ages were 66.2±13.1 years and 75.7±9.3 years in the dissection and non-dissection groups, respectively. As per our findings, nearly half (41.0%) of the patients in the dissection group were <65 years old, as were 8.3% of patients in the non-dissection group. Approximately 25% of patients in each group and subgroup were women. The rates of aortic rupture were determined to be 8.4% and 11.1% in the dissection and non-dissection groups, respectively. A degenerative etiology was observed in 74.6% and 90.1% of patients in the dissection and non-dissection groups, respectively. The mean diameters of the aortic aneurysm were 46.4±11.5 mm and 52.1±14.1 mm in the dissection and non-dissection groups, respectively. The mean diameters of saccular type aortic aneurysms were 44.9±12.1 mm and 49.2±14.3 mm in the dissection (n=377, 18.3%) and non-dissection groups (n=1,915, 54.4%), respectively (**Table 1**).

**Table 1:** Table 1 Patients’ demographics

	**Dissection**							**Non-dissection**						
	**Total**	**a) Acute**		**b) Sub-acute**		**c) Chronic**		**Total**	**Arch (Proximal: Z0-Z2)**		**Descending (Z3-Th12)**		**TAAA (Distal: L1-)**	
**Number of cases**	**2058**	**575**		**363**		**1120**		**3519**	**1492**		**1898**		**129**	
**Preoperative data**														
**Female**	515 (25.0%)	148	25.7%	92	25.3%	275	24.6%	845 (24.0%)	306	20.5%	499	26.3%	40	31.0%
**Age**														
(mean±SD)	66.2±13.1	66.0±15.3		66.0±12.4		66.3±12.1		75.7±9.3	76.1±9.1		75.3±9.5		76.9±8.7	
<65	844 (41.0%)	244	42.4%	157	43.3%	443	39.6%	294 (8.3%)	116	7.8%	168	8.9%	10	7.8%
65–74	612 (29.7%)	144	25.0%	100	27.5%	368	32.9%	1065 (30.2%)	426	28.6%	612	32.2%	27	20.9%
75–84	466 (22.6%)	116	20.2%	91	25.1%	259	23.1%	1699 (48.2%)	744	49.9%	882	46.5%	73	56.6%
85≤	136 (6.6%)	71	12.3%	15	4.1%	50	4.5%	461 (13.1%)	206	13.8%	236	12.4%	19	14.7%
**Rupture**														
Rupture (+)	173 (8.4%)	138	24.0%	9	2.5%	26	2.3%	390 (11.0%)	136	9.1%	235	12.4%	19	14.7%
Rupture (−)	1878 (91.2%)	432	75.1%	354	97.5%	1092	97.5%	3113 (88.4%)	1352	90.6%	1651	87.0%	110	85.3%
Missing	7 (0.3%)	5	0.9%	0	0.0%	2	0.2%	16 (0.4%)	4	0.3%	12	0.6%	0	0.0%
‹Type B dissection›														
Rupture (+)	163 (9.1%)	130	22.6%	9	2.5%	24	2.1%							
Rupture (−)	1606 (90.4%)	348	60.5%	304	83.7%	954	85.2%							
Missing	7 (0.3%)	5	0.9%	0	0.0%	2	0.2%							
**Stanford classification**														
Type A	282 (13.7%)	92	16.0%	50	13.8%	140	12.5%							
Type B	1776 (86.3%)	483	84.0%	313	86.2%	980	87.5%							
**Complication of aortic dissection**													
Aortic dilatation	520 (25.2%)	54	9.4%	108	29.8%	358	32.0%							
Impending rupture	93 (4.5%)	54	9.4%	17	4.7%	22	2.0%							
Rupture	182 (8.8%)	146	25.4%	9	2.5%	27	2.4%							
Malperfusion	249 (12.1%)	187	32.5%	33	9.1%	29	2.6%							
**Condition of the dissected lumen**													
Patent (double barrel)	1307 (63.5%)	377	65.6%	202	55.6%	728	65.0%							
Thrombosed	261 (12.6%)	94	16.3%	33	9.1%	134	12.0%							
Ulcer-like projection	490 (23.8%)	104	18.1%	128	35.3%	258	23.0%							
**Pathogenesis**														
Degenerative	1536 (74.6%)	386	67.1%	273	75.2%	877	78.3%	3172 (90.1%)	1382	92.6%	1668	87.9%	122	94.6%
Inflammation	2 (0.1%)	1	0.2%	0	0.0%	1	0.1%	12 (0.3%)	4	0.3%	7	0.4%	1	0.8%
Aortitis	1 (0.0%)	0	0.0%	0	0.0%	1	0.1%	5 (0.1%)	0	0.0%	4	0.2%	1	0.8%
Infection	8 (0.3%)	2	0.3%	3	0.8%	3	0.3%	89 (2.5%)	28	1.9%	59	3.1%	2	1.6%
Connective tissue disorders	30 (1.4%)	2	0.3%	11	3.0%	17	1.5%	9 (0.2%)	3	0.2%	6	0.3%	0	0.0%
Others	481 (23.3%)	184	32.0%	76	20.9%	221	19.7%	232 (6.5%)	75	5.0%	154	8.1%	3	2.3%
**Comorbidities**														
COPD	338 (16.4%)	69	12.0%	52	14.3%	217	19.4%	950 (27.0%)	420	28.2%	476	25.1%	54	41.9%
Hypertension	1689 (82.0%)	422	73.4%	296	81.5%	971	86.7%	2720 (77.2%)	1175	78.8%	1441	75.9%	104	80.6%
Cerebrovascular disease	182 (8.8%)	44	7.7%	29	8.0%	109	9.7%	468 (13.3%)	219	14.7%	234	12.3%	15	11.6%
Liver dysfunction	69 (3.3%)	38	6.6%	10	2.8%	21	1.9%	65 (1.8%)	28	1.9%	32	1.7%	5	3.9%
Hemodialysis	41 (1.9%)	12	2.1%	9	2.5%	20	1.8%	133 (3.7%)	48	3.2%	76	4.0%	9	7.0%
Carotid artery diseases	35 (1.7%)	9	1.6%	6	1.7%	20	1.8%	110 (3.1%)	57	3.8%	48	2.5%	5	3.9%
Coronary heart disease	134 (6.5%)	29	5.0%	18	5.0%	87	7.8%	565 (16.0%)	239	16.0%	302	15.9%	24	18.6%
History of thoracic surgery	214 (10.4%)	33	5.7%	29	8.0%	152	13.6%	263 (7.4%)	62	4.2%	184	9.7%	17	13.2%
History of abdominal surgery	75 (3.6%)	19	3.3%	5	1.4%	51	4.6%	294 (8.3%)	103	6.9%	169	8.9%	22	17.1%
History of thoracotomy	221 (10.7%)	33	5.7%	31	8.5%	157	14.0%	320 (9.0%)	93	6.2%	211	11.1%	16	12.4%
Marfan syndrome	29 (1.4%)	7	1.2%	6	1.7%	16	1.4%	10 (0.2%)	1	0.1%	9	0.5%	0	0.0%
**Renal function**														
Creatinine (mean±SD) (mg/dL)	1.16±1.12	1.26±1.19		1.11±1.13		1.12±1.08		1.28±1.33	1.24±1.29		1.29±1.32		1.59±1.77	
<1.2	1566 (76.0%)	392	68.2%	300	82.6%	874	78.0%	2559 (72.7%)	1089	73.0%	1384	72.9%	86	66.7%
<1.6	283 (13.7%)	96	16.7%	36	9.9%	151	13.5%	485 (13.7%)	220	14.7%	253	13.3%	12	9.3%
<2.0	83 (4.0%)	38	6.6%	9	2.5%	36	3.2%	175 (4.9%)	74	5.0%	95	5.0%	6	4.7%
2.0≤	110 (5.3%)	47	8.2%	16	4.4%	47	4.2%	285 (8.1%)	97	6.5%	163	8.6%	25	19.4%
Missing	16 (0.7%)	2	0.3%	2	0.6%	12	1.1%	15 (0.4%)	12	0.8%	3	0.2%	0	0.0%
eGFR (mean±SD)	61.1±26.3	58.9±35.1		64.6±23.7		61.0±21.3		55.8±22.3	56.2±21.0		55.8±23.2		49.8±23.5	
<15	61 (2.9%)	24	4.2%	11	3.0%	26	2.3%	159 (4.5%)	54	3.6%	95	5.0%	10	7.8%
<30	85 (4.1%)	42	7.3%	6	1.7%	37	3.3%	251 (7.1%)	94	6.3%	134	7.1%	23	17.8%
<45	322 (15.6%)	106	18.4%	49	13.5%	167	14.9%	604 (17.1%)	261	17.5%	329	17.3%	14	10.9%
<60	541 (26.2%)	152	26.4%	85	23.4%	304	27.1%	1015 (28.8%)	446	29.9%	531	28.0%	38	29.5%
60≤	1031 (50.1%)	249	43.3%	209	57.6%	573	51.2%	1467 (41.6%)	623	41.8%	801	42.2%	43	33.3%
Missing	18 (0.8%)	2	0.3%	3	0.8%	13	1.2%	23 (0.6%)	14	0.9%	8	0.4%	1	0.8%
**Diameter of the proximal fixation**													
(mean±SD) (mm)	30.3±4.8	30.2±5.0		30.2±4.8		30.4±4.7		31.2±5.0	33.0±4.7		29.9±4.9		30.0±4.2	
<20	12 (0.5%)	6	1.0%	1	0.3%	5	0.4%	24 (0.6%)	5	0.3%	19	1.0%	0	0.0%
<25	182 (8.8%)	53	9.2%	34	9.4%	95	8.5%	237 (6.7%)	39	2.6%	181	9.5%	17	13.2%
<30	697 (33.8%)	186	32.3%	126	34.7%	385	34.4%	1005 (28.5%)	261	17.5%	702	37.0%	42	32.6%
<35	822 (39.9%)	238	41.4%	147	40.5%	437	39.0%	1401 (39.8%)	647	43.4%	701	36.9%	53	41.1%
<40	267 (12.9%)	69	12.0%	43	11.8%	155	13.8%	674 (19.1%)	432	29.0%	226	11.9%	16	12.4%
40 and above	78 (3.7%)	23	4.0%	12	3.3%	43	3.8%	178 (5.0%)	108	7.2%	69	3.6%	1	0.8%
**Length of the proximal fixation**														
(mean±SD) (mm)	35.2±29.3	34.8±35.6		34.5±29.4		35.6±25.5		36.5±27.0	28.9±21.3		41.5±29.0		51.2±32.4	
<20 mm	255 (12.3%)	86	15.0%	49	13.5%	120	10.7%	381 (10.8%)	249	16.7%	131	6.9%	1	0.8%
<30 mm	761 (36.9%)	233	40.5%	142	39.1%	386	34.5%	1184 (33.6%)	717	48.1%	454	23.9%	13	10.1%
<40 mm	481 (23.3%)	129	22.4%	78	21.5%	274	24.5%	814 (23.1%)	295	19.8%	486	25.6%	33	25.6%
<50 mm	202 (9.8%)	46	8.0%	33	9.1%	123	11.0%	393 (11.1%)	103	6.9%	266	14.0%	24	18.6%
50 mm and above	359 (17.4%)	81	14.1%	61	16.8%	217	19.4%	747 (21.2%)	128	8.6%	561	29.6%	58	45.0%
**Aneurysm/aortic diameter**														
Total (mean±SD) (mm)	46.4±11.5	42.1±12.1		42.9±9.8		49.7±10.6		52.1±14.1	52.6±13.7		51.3±14.4		57.8±13.0	
Saccular (mean±SD) (mm)	44.9±12.1	44.6±16.3		42.5±11.2		45.6±10.9		49.2±14.3	50.1±14.1		48.1±14.6		52.1±11.2	

SD: standard deviation; COPD: chronic obstructive pulmonary disease; eGFR: estimated glomerular filtration rate

### Intraoperative data

Emergent operations were performed for 24.8% and 15.2% of patients in the dissection and non-dissection groups, respectively. The rate of emergent operations was determined to be high in the acute dissection subgroup (77.4%). TEVAR with bypasses was performed for approximately 10% of patients in both groups and was more frequently required in the arch (21.6%) and TAA A (14.0%) subgroups. Bypasses were required in 20.2% and 24.8% of patients in the dissection and non-dissection groups, respectively. The rate of bypasses was highest in the arch (53.5%) and TAA A (17.1%) subgroups.

The intraoperative mortality rate was 0.3% in both groups. The rates of intraoperative vascular injury were determined to be 1.7% and 3.3% in the dissection and non-dissection groups, respectively. The intraoperative rupture rate was 0.3% in the dissection group, whereas for the non-dissection group, it was 0.5% (**Table 2**).

**Table 2:** Table 2 Intraoperative data

	**Dissection**							**Non-dissection**						
	**Total (n=2058)**	**a) Acute**		**b) Sub-acute**		**c) Chronic**		**Total (n=3519)**	**Arch (Proximal: Z0-Z2)**		**Descending (Z3-Th12)**		**TAAA (Distal: L1-)**	
**Postoperative data**														
**Emergent operation**	511 (24.8%)	445	77.4%	27	7.4%	39	3.5%	537 (15.2%)	182	12.2%	333	17.5%	22	17.1%
**TEVAR procedure**														
Regular stent graft	1782 (86.5%)	521	90.6%	313	86.2%	948	84.6%	2860 (81.2%)	933	62.5%	1835	96.7%	92	71.3%
TEVAR with bypasses	187 (9.0%)	42	7.3%	33	9.1%	112	10.0%	383 (10.8%)	323	21.6%	42	2.2%	18	14.0%
Chimney TEVAR	25 (1.2%)	5	0.9%	4	1.1%	16	1.4%	76 (2.1%)	68	4.6%	4	0.2%	4	3.1%
Fenestrated stent graft	73 (3.5%)	7	1.2%	13	3.6%	53	4.7%	236 (6.7%)	206	13.8%	14	0.7%	16	12.4%
Others	4 (0.1%)	1	0.2%	0	0.0%	3	0.3%	10 (0.2%)	1	0.1%	7	0.4%	2	1.6%
**Anesthesia**														
General	1999 (97.1%)	553	96.2%	358	98.6%	1088	97.1%	3410 (96.9%)	1474	98.8%	1816	95.7%	120	93.0%
Epidural	0 (0.0%)	0	0.0%	0	0.0%	0	0.0%	5 (0.1%)	2	0.1%	3	0.2%	0	0.0%
Local	56 (2.7%)	22	3.8%	5	1.4%	29	2.6%	99 (2.8%)	15	1.0%	77	4.1%	7	5.4%
Others	3 (0.1%)	0	0.0%	0	0.0%	3	0.3%	5 (0.1%)	1	0.1%	2	0.1%	2	1.6%
**Proximal landing condition**														
Elephant trunk	94 (4.5%)	13	2.3%	10	2.8%	71	6.3%	123 (3.5%)	26	1.7%	95	5.0%	2	1.6%
Graft	234 (11.3%)	37	6.4%	35	9.6%	162	14.5%	286 (8.1%)	85	5.7%	191	10.1%	10	7.8%
Native aorta	1730 (84.0%)	525	91.3%	318	87.6%	887	79.2%	3110 (88.3%)	1381	92.6%	1612	84.9%	117	90.7%
**Endoleaks**														
Type 1	114 (5.5%)	28	4.9%	18	5.0%	68	6.1%	144 (4.0%)	83	5.6%	54	2.8%	7	5.4%
Type 2	35 (1.7%)	12	2.1%	5	1.4%	18	1.6%	46 (1.3%)	15	1.0%	24	1.3%	7	5.4%
Type 3	13 (0.6%)	1	0.2%	0	0.0%	12	1.1%	14 (0.4%)	2	0.1%	9	0.5%	3	2.3%
Type 4	24 (1.1%)	5	0.9%	3	0.8%	16	1.4%	79 (2.2%)	28	1.9%	43	2.3%	8	6.2%
Not available	31 (1.5%)	18	3.1%	4	1.1%	9	0.8%	20 (0.5%)	6	0.4%	13	0.7%	1	0.8%
**Additional procedures**	248 (12.0%)	83	14.4%	37	10.2%	128	11.4%	440 (12.5%)	307	20.6%	105	5.5%	28	21.7%
Occlusion of the aortic branches (*)	475 (23.0%)	127	22.1%	76	20.9%	272	24.3%	919 (26.1%)	775	51.9%	100	5.3%	44	34.1%
Brachiocephalic artery	6 (0.2%)	1	0.2%	0	0.0%	5	0.4%	35 (0.9%)	33	2.2%	2	0.1%	0	0.0%
Left common carotid artery	66 (3.2%)	14	2.4%	15	4.1%	37	3.3%	213 (6.0%)	210	14.1%	3	0.2%	0	0.0%
Left subclavian artery	449 (21.8%)	122	21.2%	70	19.3%	257	22.9%	831 (23.6%)	772	51.7%	59	3.1%	0	0.0%
Celiac artery	20 (0.9%)	1	0.2%	4	1.1%	15	1.3%	80 (2.2%)	0	0.0%	38	2.0%	42	32.6%
Superior mesenteric artery	8 (0.3%)	3	0.5%	1	0.3%	4	0.4%	21 (0.6%)	0	0.0%	5	0.3%	16	12.4%
Renal artery	7 (0.3%)	2	0.3%	1	0.3%	4	0.4%	18 (0.5%)	0	0.0%	3	0.2%	15	11.6%
Bypasses (*)	417 (20.2%)	79	13.7%	73	20.1%	265	23.7%	873 (24.8%)	798	53.5%	53	2.8%	22	17.1%
Aorta-brachiocephalic artery	6 (0.2%)	4	0.7%	0	0.0%	2	0.2%	53 (1.5%)	51	3.4%	2	0.1%	0	0.0%
Aorta-left common carotid artery	8 (0.3%)	4	0.7%	0	0.0%	4	0.4%	52 (1.4%)	50	3.4%	2	0.1%	0	0.0%
Aorta-left subclavian artery	7 (0.3%)	3	0.5%	0	0.0%	4	0.4%	54 (1.5%)	50	3.4%	4	0.2%	0	0.0%
Carotid-carotid artery	8 (0.3%)	3	0.5%	1	0.3%	4	0.4%	31 (0.8%)	31	2.1%	0	0.0%	0	0.0%
Carotid-left subclavian artery	215 (10.4%)	41	7.1%	39	10.7%	135	12.1%	357 (10.1%)	348	23.3%	9	0.5%	0	0.0%
Subclavian-subclavian artery	229 (11.1%)	43	7.5%	41	11.3%	145	12.9%	574 (16.3%)	543	36.4%	31	1.6%	0	0.0%
Celiac artery	8 (0.3%)	0	0.0%	2	0.6%	6	0.5%	17 (0.4%)	0	0.0%	5	0.3%	12	9.3%
Superior mesenteric artery	12 (0.5%)	2	0.3%	3	0.8%	7	0.6%	30 (0.8%)	0	0.0%	8	0.4%	22	17.1%
Renal artery	9 (0.4%)	0	0.0%	2	0.6%	7	0.6%	19 (0.5%)	0	0.0%	5	0.3%	14	10.9%
Others	21 (1.0%)	2	0.3%	2	0.6%	17	1.5%	82 (2.3%)	75	5.0%	5	0.3%	2	1.6%
**Vascular injury**	36 (1.7%)	7	1.2%	7	1.9%	22	2.0%	119 (3.3%)	54	3.6%	58	3.1%	7	5.4%
**Aneurysm rupture**	8 (0.3%)	6	1.0%	0	0.0%	2	0.2%	20 (0.5%)	6	0.4%	14	0.7%	0	0.0%
**Intraoperative radiation dose**														
(mean±SD) (mGy)	23.9±25.1	24.9±25.2		20.5±17.3		24.6±27.0		25.0±31.8	28.3±25.4		21.4±35.4		40.4±32.4	
**Intraoperative death**	8 (0.3%)	7	1.2%	0	0.0%	1	0.1%	13 (0.3%)	4	0.3%	8	0.4%	1	0.8%

TEVAR: thoracic endovascular aortic repair; SD: standard deviation; (*): there are some overlapping.

### Postoperative data

The in-hospital mortality rates (including intraoperative death) were 3.6% and 4.4% in the dissection and non-dissection groups, respectively. The mortality rates of the acute (10.0%) and TAA A subgroups (11.6%) were deemed to be the highest. Renal insufficiency occurred in 8.7% and 13.2% of patients in the acute dissection and TAA A subgroups, respectively. Cerebrovascular damage occurred in 8.5% of patients in the arch subgroup. Multiple organ failure and hemodialysis occurred in 7.0% and 6.2% of patients, respectively, in the TAA A subgroup. The overall rates of paraplegia were 2.2% and 4.8% in the dissection and non-dissection groups, respectively. The rates of paraplegia were 4.5% and 2.3% in the acute dissection and TAA A subgroups, respectively.

Endoleak data were obtained for 5,037 patients for whom contrast-enhanced computed tomography (CT) was performed during their hospital stay. The number of type 1 and 3 endoleaks at hospital discharge was 124 (6.5%) and 157 (4.9%) in the dissection and non-dissection groups, respectively. The rates of type 1 endoleak were found to be 6.9% and 7.2% in the chronic dissection and arch subgroups, respectively. The rates of type 2 endoleak were 5.7% and 5.9% in the sub-acute dissection and TAA A subgroups, respectively (**Table 3**).

**Table 3:** Table 3 Postoperative data

	**Dissection**							**Non-dissection**						
	**Total**	**a) Acute**		**b) Sub-acute**		**c) Chronic**		**Total**	**Arch (Proximal: Z0-Z2)**		**Descending (Z3-Th12)**		**TAAA (Distal: L1-)**	
**Data at hospital discharge**														
**Number of cases (alive)**	2050	568		363		1119		3506	1488		1890		128	
**Duration of hospitalization after the operation**														
(median (IQR))	11 (8–20)	20 (11–34)		11 (8–17)		9 (7–14)		11 (7–20)	12 (8–22)		10 (7–18)		14 (8–26)	
**Complications**														
Thromboembolism	16 (0.7%)	8	1.4%	0	0.0%	8	0.7%	39 (1.1%)	21	1.4%	14	0.7%	4	3.1%
Renal insufficiency	89 (4.3%)	50	8.7%	9	2.5%	30	2.7%	162 (4.6%)	74	5.0%	71	3.7%	17	13.2%
Hemodialysis introduction	35 (1.7%)	25	4.3%	5	1.4%	5	0.4%	61 (1.7%)	31	2.1%	22	1.2%	8	6.2%
Cerebrovascular damage	56 (2.7%)	20	3.5%	10	2.8%	26	2.3%	167 (4.7%)	127	8.5%	39	2.1%	1	0.8%
Paraplegia	45 (2.2%)	26	4.5%	4	1.1%	15	1.3%	109 (3.1%)	38	2.5%	68	3.6%	3	2.3%
Multiple organ failure	40 (1.9%)	33	5.7%	2	0.6%	5	0.4%	56 (1.6%)	23	1.5%	24	1.3%	9	7.0%
Aneurysm rupture	10 (0.4%)	5	0.9%	0	0.0%	5	0.4%	19 (0.5%)	7	0.5%	11	0.6%	1	0.8%
Additional procedures	52 (2.5%)	26	4.5%	8	2.2%	18	1.6%	95 (2.7%)	58	3.9%	31	1.6%	6	4.7%
**In-hospital death (post-operation)**	67 (3.2%)	51	8.9%	3	0.8%	13	1.2%	142 (4.0%)	58	3.9%	70	3.7%	14	10.9%
**In-hospital death (intra- and post-operation)**	75 (3.6%)	58	10.0%	3	0.8%	14	1.2%	155 (4.4%)	62	4.1%	78	4.1%	15	11.6%
**Data of imaging modalities at discharge**														
**Number of cases**	1882	513		349		1020		3155	1349		1687		119	
**Stent graft migration**	2 (0.1%)	1	0.2%	0	0.0%	1	0.1%	0 (0.0%)	0	0.0%	0	0.0%	0	0.0%
**Endoleaks at discharge**														
Type 1	107 (5.6%)	22	4.3%	15	4.3%	70	6.9%	131 (4.1%)	97	7.2%	29	1.7%	5	4.2%
Type 2	91 (4.8%)	19	3.7%	20	5.7%	52	5.1%	124 (3.9%)	63	4.7%	54	3.2%	7	5.9%
Type 3	17 (0.9%)	4	0.8%	2	0.6%	11	1.1%	26 (0.8%)	10	0.7%	11	0.7%	5	4.2%
Type 4	6 (0.3%)	1	0.2%	1	0.3%	4	0.4%	44 (1.3%)	20	1.5%	18	1.1%	6	5.0%
Not available	103 (5.4%)	41	8.0%	17	4.9%	45	4.4%	198 (6.2%)	73	5.4%	118	7.0%	7	5.9%

IQR: interquartile range

### Causes of death

Intraoperative rupture, hemorrhage, or systemic circulatory failure were the most common causes of intraoperative death, accounting for 6 and 10 patients in the dissection and non-dissection groups, respectively. During hospital admission, vascular-related complications (including retrograde type A aortic dissection [RTAD]) were identified to be the most common causes of death in the dissection group. Meanwhile, in the non-dissection group, infection and sepsis were the most common causes of postoperative death (**Table 4**).

**Table 4:** Table 4 Causes of death

**Causes of death**	**Dissection**	**Non-dissection**
**[During the operation]**		
Rupture/hemorrhage/systemic circulatory failure	6	10
Malperfusion/multiple organ failure (MOF)	1	0
Retrograde type A aortic dissection (RTAD)	0	1
Intestinal necrosis	1	1
Antipiracy of contrast agents	0	1
**TOTAL**	**8**	**13**
**[In-hospital (after the operation)]**		
Rupture/hemorrhage	10	19
Malperfusion/multiple organ failure	3	0
Cerebrovascular damage	4	12
Multiple thromboembolism	0	1
Arrhythmia/low output syndrome/heart failure	7	15
Pneumonia/respiratory failure	8	21
Liver failure	0	2
Renal failure	1	7
Intestinal necrosis/enterocolitis	2	6
Vascular-related complications (including RTAD)	17	19
Infection/sepsis	11	26
DIC	3	5
Cancer	0	6
Multiple injury (trauma)	1	1
Unknown	0	2
**TOTAL**	**67**	**142**

DIC: disseminated intravascular coagulation

## Discussion

The JACSM registry began in July 2006 after the approval of the use of stent graft device in Japan, and the data input and storage have been transferred from the JACSM database to the National Clinical Database (NCD) since January 2016. The 2016 annual data was reported on the JACSM website, and the committee has decided to publish the annual data henceforth. These data can help with preoperative planning for thoracic aortic diseases.

In this study, patients were divided into dissection and non-dissection groups as TEVAR is performed for aortic dissection as well as entry/re-entry closure. The dissection group was categorized according to the timing of the operation: acute (TEVAR within 2 weeks of onset), sub-acute (TEVAR between 2 weeks and 2 months of onset), and chronic (TEVAR more than 2 months after onset). The threshold of 2 months was chosen according to the Japanese Circulation Society (JCS) guidelines published in 2006.^5)^ However, the sub-acute category was not included in the JCS guideline in 2011,^2)^ and the threshold was then revised from 2 months to 3 months in the 2020 JCS guideline. To account for the revised guidelines, the JACSM-NCD registry will be adjusted to include an input for day of onset so that the interval between onset and TEVAR can be automatically calculated.

Patients in the acute dissection subgroup were determined to have a high mortality rate (10.0%), which may be due to the high rate of emergent operations (77.4%) and ruptures (24.0%) in this subgroup. This was in contrast to the mortality rates of the sub-acute (0.8%) and chronic (1.2%) dissection subgroups, which were found to be lower, as were their emergent operation rates (7.4% and 3.5%, respectively). Furthermore, the mean aneurysm diameters of the sub-acute (42.9 mm) and chronic (49.7 mm) dissection groups were below the threshold (55 mm to 60 mm: Class IIa) in the guidelines. These data indicate that a considerable number of TEVAR procedures were performed preemptively for the closure of the entry of the dilating false lumen or to prevent an aortic rupture. These indications for operation cannot be distinguished in the current JACSM registry; therefore, a new input for the indication for TEVAR will be included after 2021.

Vascular-related complications have been identified as the most common cause of postoperative death during admission in the dissection group, including RTAD. The reporting of RTAD will also be added to the registry after 2021.

The non-dissection group was divided into three categories according to the stent graft placement location, as described in the Japan Adult Cardiovascular Surgery Database: arch (zone 0 to 2), descending (zone 3 to Th 12), and TAA A (L1 and distal). Stents were either placed in the arch, descending aorta, and TAA A at a rate of 42.4%, 53.9%, and 3.6%, respectively, which differed from the rates presented in the JACSM database (62.4%, 27.0%, and 10.6%, respectively).^6)^ This may be due to the fact that TEVAR is not the standard treatment for patients in the arch or TAA A subgroups. Because the mean age and demographics of patients in the arch subgroup were similar to those of patients in the descending subgroup in this study, the indication for TEVAR was likely based on the anatomical suitability and policies of each facility, not on the patients’ comorbidities. In contrast, the mean age and risks of the patients in the TAA A subgroup were deemed higher than those of the patients in the descending subgroup, indicating that TEVAR may be reserved for high risk patients in this subgroup, which may account for the high mortality rate among these patients.

Patients in the arch subgroup had a higher rate of cerebrovascular damage than those in the descending or TAA A subgroup; however, the rate of paraplegia was determined to be similar among the three subgroups. Renal dysfunction was highest in the TAA A category, suggesting that a main cause of paraplegia may be shower embolisms or length of stent graft coverage, not the location of the stent graft.

Type 1 and 3 endoleaks are critical adverse events that can be treated. The high rate of type 1 endoleaks in the arch subgroup may be attributed to a poor fit of the proximal end of the stent graft along the arch (as seen by a beak sign on CT scan). Similarly, the high rate of type 3 endoleaks in the TAA A subgroup may be due to a poor fit of the stent graft at the angulated junction of the aorta and the phrenic arteries.

TEVAR was used effectively to treat patients with thoracic aortic disease in Japan in 2017. Patients with an acute dissection, or those with stents placed in the arch or TAA A, can have a high risk of mortality and complications. Furthermore, the rate of type 1 and type 3 endoleaks is high. Thus, close and careful follow-up, especially in patients with acute dissections or requiring stents in the arch or TAA A, is necessary to improve the outcomes of TEVAR.

## Notes

*The JACSM collaborators are listed in the Acknowledgements.
